# Supplementary material for: Polycyclic Aromatic Hydrocarbons in Sediments/Soils of the Rapidly Urbanized Lower Reaches of the River Chaohu, China
Source: Int J Environ Res Public Health. 2019 Jun 28;16(13):2302. doi: 10.3390/ijerph16132302 (PMC6651651; doi:10.3390/ijerph16132302)
Supplement: Supplementary file 1 [file ijerph-16-02302-s001.pdf]

International Journal of
Environmental Research and Public Health

MDPI

Article

Polycyclic Aromatic Hydrocarbons in Sediments/Soils of the Rapidly Urbanizing Lower Reaches of the River Chaohu, China

Huanling Wu [1,2], Binghua Sun [1] and Jinhua Li [1,3,*]

[1] School of Resources and Environmental Engineering, Anhui University Anhui, Hefei 230601, Anhui, China
[2] Key laboratory of Aqueous Environment Protection and Pollution Control of Yangtze River in Anhui of Anhui Provincial Education, Anqing Normal University, Anqing 246001, Anhui, China
[3] School of Life Science, Hefei Normal University, Hefei 230601, Anhui, China
* Correspondence: jhli@ahu.edu.cn

Supplementary material:

Table S1. Information on the target analyte, abbreviation, CAS (chemical abstracts service) number, quantification ion, toxic equivalent factor, effect range low, effects range median value, and equilibrium sediment benchmark.

Compound	Abbreviation	CAS	Quant. Ion [a]	TEF [b]	EML [c]	EMR [d]	ESB [e]
Naphthalene	Nap	91–20–3	126	0.001	160	2100	385
Acenaphthylene	Acy	208–96–8	152	0.001	44	640	452
Acenaphthene	Acp	83–32–9	153	0.001	16	500	491
Fluorene	FLR	86–73–7	165	0.001	19	540	538
Phenanthrene	PHE	85–01–8	178	0.001	240	1500	596
Anthracene	Ant	120–12–7	178	0.01	853	1100	594
Fluranthrene	FLT	206–44–0	202	0.001	600	5100	707
Pyrene	PYR	129–00–0	202	0.001	665	2600	697
Benzo[a] anthracene	BaA	56–55–3	228	0.1	261	1600	841
Chrysene	CHR	218–01–9	228	0.01	384	2800	844
Benzo[b]fluoranthene	BbF	205–99–2	252	0.1	NA	NA	979
Benzo[k]fluoranthene	BkF	207–08–9	252	0.1	NA	NA	981
Benzo[a]pyrene	BaP	50–32–8	252	1	430	1600	965
Indo[1,2,3-c,d]pyrene	IcP	193–39–5	278	0.1	NA	NA	1115
Dibenzo[a,h]anthracene	DhA	53–70–3	276	1	63.4	260	1123
Benzo[g,h,i]perylene	BgP	191–24–2	278	0.01	NA	NA	1094

[a] Quantification ion (m/z, Dalton); [b] Toxic equivalent factor obtained from the literature (Nisbet & LaGoy, 1992); [c] Effects range obtained from the literature (Qiao et al., 2006); [d] Effects range median value obtained from the literature (Qiao et al., 2006); [e] Equilibrium sediment benchmark (μg g^{-1} OC). The data was obtained from previous work (Mehler et al. 2011, Wang et al. 2011).
